# Video-based estimation of blood pressure

**DOI:** 10.1371/journal.pone.0311654

**Published:** 2025-01-30

**Authors:** Ananyananda Dasari, Laszlo A. Jeni, Conrad S. Tucker

**Affiliations:** 1 Department of Mechanical Engineering, Carnegie Mellon University, Pittsburgh, PA, United States of America; 2 The Robotics Institute, Carnegie Mellon University, Pittsburgh, PA, United States of America; Maulana Abul Kalam Azad University of Technology West Bengal, INDIA

## Abstract

In this work, we propose a non-contact video-based approach that estimates an individual’s blood pressure. The estimation of blood pressure is critical for monitoring hypertension and cardiovascular diseases such as coronary artery disease or stroke. Estimation of blood pressure is typically achieved using contact-based devices which apply pressure on the arm through a cuff. Such contact-based devices are cost-prohibitive as well as limited in their scalability due to the requirement of specialized equipment. The ubiquity of mobile phones and video-based capturing devices motivates the development of a non-contact blood pressure estimation method—Video-based Blood Pressure Estimation (V-BPE). We leverage the time difference of the blood pulse arrival at two different locations in the body (Pulse Transit Time) and the inverse relation between the blood pressure and the velocity of blood pressure pulse propagation in the artery to analytically estimate the blood pressure. Through statistical hypothesis testing, we demonstrate that Pulse Transit Time-based approaches to estimate blood pressure require knowledge of subject specific blood vessel parameters, such as the length of the blood vessel. We utilize a combination of computer vision techniques and demographic information (such as the height and the weight of the subject) to capture and incorporate the aforementioned subject specific blood vessel parameters into our estimation of blood pressure. We demonstrate the robustness of V-BPE by evaluating the efficacy of blood pressure estimation in demographically diverse, outside-the-lab conditions. V-BPE is advantageous in three ways; 1) it is non-contact-based, reducing the possibility of infection due to contact 2) it is scalable, given the ubiquity of video recording devices and 3) it is robust to diverse demographic scenarios due to the incorporation of subject specific information.

## Introduction

The cardiovascular system, comprising primarily of the heart and the blood vessels, is responsible for regulating the flow of blood to all organs of the body through the periodic pumping action of the heart [1]. The pumping action of the heart causes the circulating blood to exert a force on the walls of the arteries, which is termed as the blood pressure [1–3]. Sustaining adequate blood pressure in a narrow range is vital to the transport of nutrients and oxygen through the blood to various organs of the body [2]. The narrow range of blood pressure is maintained through the interplay of various physiological processes such as the cardiac output, peripheral resistance and reflex mechanisms [3, 4]. Impairment in the aforementioned physiological processes due to disease causes either higher-than-adequate blood pressure (hypertension) or lower-than-adequate blood pressure (hypotension) [5, 6]. Hypertension increases the risk of heart attack and stroke, as well as cardiovascular and kidney diseases [3–5]. Hypotension impedes oxygen transport to the organs, causing fatigue and dizziness [3, 4, 6]. Blood pressure levels are influenced by multiple factors, such as cardiovascular health, age, lifestyle, environment and genetics highlighting the importance of blood pressure estimation [7].

Blood pressure is typically reported as two values—the systolic blood pressure (the maximum pressure exerted by the heart during a pulse) and the diastolic blood pressure (the pressure in the arteries during the time between consequent pulses) [8]. The exact values of systolic and diastolic blood pressure are measured in clinical settings through the utilization of an intra-arterial catheter invasively placed in various arteries and connected to a pressure transducer [9]. The invasive nature of the intra-arterial catheter increases the risk of infection during blood pressure monitoring and is not implementable in non-clinical settings [9]. The auscultatory method utilizes a non-invasive, contact-based mercury sphygmomanometer (blood pressure measurement device) and a stethoscope and has been the gold standard for blood pressure estimation [10, 11]. However, the auscultatory method also carries the risk of infection through contact and requires trained medical personnel to estimate blood pressure, reducing its practicality in non-clinical settings [11]. Also, the growing concern over mercury-based devices has reduced the utilization of the auscultatory method in blood pressure measurement [10]. The oscillometric method detects pressure oscillations through a cuff placed on the subject’s upper arm and estimates the blood pressure through an empirical algorithm [9, 10]. The oscillometric method is now accepted as the gold standard for the measurement of blood pressure [9, 10]. Such cuff-based contact-based devices are cost-prohibitive and carry the risk of infection, necessitating sanitizing procedures. Cuff-less blood pressure estimation devices utilize the electrocardiogram (ECG) or the photoplethysmograph (PPG) signals to calculate the signal correlation which provides an estimate of the blood pressure [12–14]. The ECG signal is measured using a Holter monitor which detects electrical disturbances under the skin corresponding to the pulsations of the heart [15]. PPG signals are detected using light intensity oscillations transmitted through the skin, which correspond to the heart beat [16]. Non-contact blood pressure estimation approaches have focused on analyzing the recorded ECG and PPG signals to calculate the blood pressure. Such methods reduce the requirement for contact-based sensors by utilizing remote photoplethysmography (rPPG) to estimate the PPG signal. However, the ECG signal still requires a contact-based lead to be affixed to the skin, limiting the scalability of such non-contact-based approaches.

In this study, we introduce V-BPE, a novel Video-based Blood Pressure Estimation method that leverages rPPG signals extracted from video recordings to estimate systolic and diastolic blood pressure. V-BPE specifically captures rPPG signals from two key anatomical locations—the face and the hand—both of which are concurrently visible within a single video frame. The face and hand are selected due to their suitability for unobstructed rPPG signal acquisition [17]. V-BPE utilizes a standard video recorded with a mobile phone/computer camera with a minimum video resolution requirement of 320 × 240 pixels and a frame rate requirement of 30 frames per second (fps).

V-BPE estimates the blood pressure based on the inverse relation between the blood pressure and the velocity of blood pressure pulse in the artery or Pulse Wave Velocity (PWV) [18]. To estimate PWV, we compute Pulse Transit Time (PTT) [19], defined as the temporal delay between rPPG signals obtained from two regions at different distances from the heart (such as the face and the hand). We demonstrate the critical role of demographic factors such as the subject’s height (measured by a medical grade height scale) in the estimation of PWV. Such demographic factors are necessary to estimate key physiological parameters such as blood vessel length and blood velocity, which are essential for blood pressure estimation. Existing video-based blood pressure estimation methods often bypass the inclusion of such demographic factors, thereby compromising robustness in diverse, real-world conditions. To this end, we create a dataset containing subject videos with ground truth blood pressure and associated demographic information. V-BPE incorporates the demographic information to compute key physiological parameters (such as blood vessel length and blood velocity) to perform robust estimation of systolic and diastolic blood pressure.

In summary, the key contribution of the current study is the development of V-BPE, a demographically robust non-contact blood pressure estimation approach. Our statistical analysis underscores the necessity of incorporating subject specific demographic data in blood pressure estimation. Additionally, we introduce a new dataset to rigorously evaluate the performance and robustness of existing video-based blood pressure estimation methods compared to V-BPE in real-world, outside-the-lab environments.

Research on the estimation of blood pressure can be classified into contact-based and non-contact-based approaches. The following sections review these approaches, highlighting the contributions of the current work.

### Contact-based blood pressure estimation

Contact-based approaches for estimating blood pressure have focused on the detection of the ECG and the PPG signals from different locations on the body and analyzing properties such as signal correlation to estimate the blood pressure. Gesche et al. developed a linear regression model to relate the systolic blood pressure with the pulse wave velocity calculated from the time difference between the ECG and finger PPG signals [13]. The study is limited to deployment in clinical settings due to the requirement for chest electrodes to detect the ECG signal. Chen et al. utilized two separate contact-based PPG signals from the ear and the toe and developed empirical relations to calculate PWV for different levels of systolic blood pressure values [20]. The study shows that different PWV values can be utilized to compute the systolic and the diastolic blood pressure values separately. However, the empirical relations developed are not generalizable to a wide range of blood pressure values, limiting their practicality. Ma developed an ECG-PTT-based blood pressure estimation method and introduced subject specific calibration [12]. The approach however requires an initial blood pressure measurement to be provided as a reference. Buxi et al. demonstrated that frequent subject specific re-calibration is necessary to ensure the accuracy of the ECG-PTT based approach to estimate blood pressure [14]. Bang et al. proposed a bio-impedance and ECG based approach to estimate the electrical signals of the blood flowing through the radial artery in the wrist [21]. However, bio-impedance signal collection is limited by the necessity of a contact wrist band integrated with electrodes to measure electrical changes in the blood, restricting their utilization due to the risk of infection. Studies have also utilized the Penaz principle which clamps the arteries in the finger with a volume clamping device to continuously monitor blood pressure [22]. Such clamping devices introduce errors due to either insufficient or excess pressure leading to incorrect blood pressure estimates. Chandrasekhar et al. modified a smartphone to apply the pressure to clamp the finger to continuously monitor blood pressure [23]. Though the smartphone integration improves the accessibility of non-clinical blood pressure estimation, the modifications require the application of external pressure limiting their use in outside-the-lab environments.

In summary, contact-based approaches have employed cuff-based pressure application methods and cuff-less ECG-PPG-based methods. ECG-PPG methods attempt to estimate the blood pressure based on empirical estimates, which do not include subject specific demographic information. Moreover, the necessity of contact between the device and the skin introduces the risk of infection and person-person transmission. Additionally, the requirement of proprietary devices such as blood pressure cuffs and ECG detecting sensors reduces the ubiquity of contact-based approaches. Such limitations motivate the development of non-contact-based approaches to estimate the blood pressure.

### Non-contact-based blood pressure estimation

Non-contact-based approaches to estimate blood pressure have explored video-based solutions due to the ubiquity of video recording devices such as computers and mobile phones. Sugita et al. explored the correlation between the PTT computed from different rPPG signals and the blood pressure [24]. Murakami et al. report a high negative correlation between PTT (obtained from PPG signals of the wrist and ankle) and blood pressure [25]. Both the studies [24, 25] establish the correlation between the PTT and the blood pressure. To estimate the value of the blood pressure, studies have utilized the Moens Korteweg equation, which relates the PWV with arterial distensibility [26]. However, the equation assumes isotropic arterial wall conditions, which are atypical of actual arterial wall properties. To account for the physical properties observed in the actual arterial wall, the Moens Korteweg equation is modified in studies by Bramwell and Hughes to include non-isotropic arterial wall conditions using data driven methods or finite element analysis [27, 28]. Though the Moens Korteweg equation is developed for a single blood vessel, it can relate the PTT (which is measured from two different blood vessels) to the blood pressure by computing the PWV as an intermediate [27–29]. Studies have shown that the modified Moens Korteweg relations are limited by the demographic variability among populations [30]. To reduce the dependency on demographic parameters, Ma et al. developed a PWV-Blood Pressure relation based on the Fung hyper-elastic model of the artery. The study relies on arterial studies performed in vitro, and does not provide an analysis of the effect of demographic variability on the estimated values of blood pressure in outside-the-lab settings. Wang et al. developed a cuff-less approach for estimating the blood pressure utilizing the finger PPG, ECG and the heart rate using a modified form of the Moens Korteweg equation [31]. Liu et al. employed multi-wavelength PPG signal detection to analyze the skin penetration depth of light at different wavelengths [32]. The wavelength dependent skin penetration depth of light computes the PTT which is utilized to estimate the mean blood pressure through an empirical relation. Hassan et al. utilized heart rate information in addition to the ECG signal and reported a strong correlation between the heart rate and the systolic blood pressure [33]. However, the study reports that the explicit determination of the heart rate to estimate the blood pressure could introduce errors due to incorrect heart rate estimation. Finnegan et al. explored a diverse set of features from ECG and PPG signals to inform blood pressure estimation [34]. The approach develops regression equations based on chemically induced blood pressure changes in the body, which is not representative of the natural blood pressure changes. Jeong et al. [35] explored the correlation between blood pressure and the PTT signal and reported that the level of correlation varied among subjects. Secerbegovic et al. [36] developed a regression-based approach to relate the variation of blood pressure with the PTT and demonstrated that regression-based approaches can inform blood pressure variation rather than the absolute value of blood pressure. Zhou et al. [37] developed a regression-based approach linking the blood pressure to the subject’s Body Mass Index (BMI) in various environemntal conditions. Hamoud et al. [38] developed a deep convolutional neural network approach to identify skin regions of interest and estimate the blood pressure from facial videos. The approach demonstrated a good correlation with additional vital signs such as respiration rate. Trirongjitmoah et al. [39] utilized a continuous wavelet transform of the PPG signal to learn the SBP and BP utilizing a deep convolutional neural network. Luo et al. [40] utilized transdermal optimal imaging to identify a blood haemoglobin signal from a video of a subject’s face. Feature engineering was employed to identify a set of features that correlate with the blood pressure. However, the approach is tested in laboratory settings and is constrained to a narrow range of blood pressure values. Such constraining is typically encountered in machine learning approaches and has been identified as a critical challenge in the estimation of blood pressure [41].

In summary, non-contact blood pressure estimation approaches have focused on the video-based identification of relevant signals such as the ECG and the PPG signals. Non-contact approaches have been limited by the demographic biases that affect the blood pressure estimation. Table 1 shows a summary of the existing studies in blood pressure estimation as compared to the current work.

**Table 1:** Summary of existing human blood pressure estimation method.

Authors	Year	Contact-based sensor (Direct BP information through contact with the skin)	Contact-based ECG (ECG signal measured to infer BP)	Non-contact-based correlation studies	Non-contact-based with rPPG extraction from single video	Outside-the-lab study (Environmental conditions are not controlled)	Blood pressure estimation (systolic-S or diastolic-D)
Hassan et al. [33]	2008		✓				S
Bang et al. [21]	2009	✓	✓				S
Gesche et al. [13]	2012	✓	✓				S
Chen et al. [20]	2012	✓					S, D
Ma et al. [12]	2014	✓	✓				S, D
Wang et al. [31]	2014	✓					S, D
Buxi et al. [14]	2015		✓				S
Jeong & Finkelstein [35]	2016			✓			S
Secerbegovic et al. [36]	2016			✓			S, D
Liu et al. [32]	2018			✓			S
Chandrasekhar et al. [23]	2018	✓					S, D
Luo et al. [40]	2019			✓	✓		S, D
Zhou et al. [37]	2019			✓	✓		S, D
Finnegan et al. [34]	2023	✓					S, D
Hamoud et al. [38]	2023			✓			S, D
Chen et al. [47]	2023			✓			S, D
Cheng et al. [48]	2023			✓			S, D
Xing et al. [49]	2023			✓			S, D
Trirongjitmoah et al. [39]	2023			✓			S, D
Current Study (V-BPE)	2023				✓	✓	S, D

In this work, we utilize an RGB camera that is a component of most mobile phones and computer-based systems. Such availability improves the accessibility of V-BPE due to the ubiquity of devices with video recording capabilities, such as smartphones or digital cameras. V-BPE utilizes a modified form of the Moens Korteweg equation to estimate the blood pressure [42, 43]. Subject specific parameters such as the lengths of blood vessels are estimated using demographic information such as the height of the subject and a full length image of the subject. Two locations—the face and the hand are selected to estimate rPPG signals [20, 44]. Dasari et al. reported that the CHROM [44] and BKF [45] algorithms demonstrate consistency of rPPG signal extraction in demographically diverse conditions [46]. Leveraging the consistency of rPPG signal extraction in demographically diverse settings, V-BPE utilizes the CHROM algorithm to extract the rPPG signal. The mean peak-peak time difference between the two signals is computed to obtain the face-to-hand PTT, which is critical for the calculation of the blood pressure using the modified Moens Korteweg equation. V-BPE computes different PTT values to independently estimate the systolic and diastolic blood pressure values. Studies by Hamoud et al. [38] Chen et al. [47], Cheng et al. [48] and Xing et al. [49] explore deep learning models such as Convolutional Neural Networks and Recurrent Neural Networks to estimate the blood pressure, utilizing information from facial videos and ECG signals. Mehta et al. have reported that deep learning approaches of estimating vital signs face challenges such as over-constraining the task (the distribution of ground truth is limited to a certain range) and data leakage (where data similar to the training data is present in the test data) [41]. Such challenges introduce bias in vital sign estimation approaches. V-BPE does not involve a data-driven training procedure, mitigating the effect of data leakage. We mitigate the effect of task over-constraining by creating the Face-Hand dataset which consists of subject videos in outside-the-lab settings with associated demographic and ground truth information. The Face-Hand dataset consists of unfiltered ground truth data from FDA-approved measurement devices and does not constrain the range of blood pressure values. V-BPE is rigorously tested on the Face-Hand dataset to assess its robustness and efficacy. Additionally, the Face-Hand dataset is employed to evaluate the robustness of existing blood pressure estimation methods, providing a comparative analysis against V-BPE. In summary, the novel contributions of this study are:

- The development and comparative evaluation of a non-contact-based approach to estimate human blood pressure, that is scalable to resource constrained environments
- The evaluation of the effect of demographic factors on the computation of pulse wave velocity for the estimation of blood pressure
- The creation of the Face-Hand dataset consisting of videos and demographic data of subjects with their face and hand in the same video frame, to facilitate PTT-based blood pressure estimation in outside-the-lab settings

## Methods

### Overview

V-BPE utilizes a pulse transit time (PTT) based approach to estimate the blood pressure rather than inducing external pressure on the artery. V-BPE utilizes a face and hand tracking algorithm to identify facial and hand landmarks of the subject in a video. The extracted facial and hand landmarks are processed by a remote-PPG algorithm to extract the PPG signal. The PTT is calculated utilizing the peak-peak difference between the two PPG signals (face PPG and hand PPG). V-BPE utilizes the calculated PTT to estimate the systolic and diastolic blood pressures. The Moens Korteweg equation [42, 43] relates the Pulse Wave Velocity (PWV) to the arterial distensibility and is given by

$$\begin{array}{c} PWV=\sqrt{\frac{Eh}{\rho D}} \end{array}$$ (1)

where *PWV* is the pulse wave velocity, *E* is the modulus of elasticity of the artery, *h* is the thickness of the artery wall, *ρ* is the density of blood and *D* is the arterial diameter. The modulus of elasticity, *E* can be expressed in terms of the blood pressure as follows [28].

$$\begin{array}{c} E=E_{0}e^{\alpha BP} \end{array}$$ (2)

where *E*_0_ is the zero pressure modulus of elasticity of the arterial wall, *α* is a constant and *BP* is the blood pressure. From Eqs 1 and 2, we obtain the following relation for the PWV.

$$\begin{array}{c} PWV^{2}=\frac{hE_{0}e^{\alpha BP}}{\rho D} \end{array}$$ (3)

The PWV can be computed from the PTT by utilizing the difference (*L*) in the lengths of the artery at the two locations [29, 31, 50].

$$\begin{array}{c} PWV=\frac{L}{PTT} \end{array}$$ (4)

From Eqs 3 and 4, we obtain the following relation between PWV and BP

$$\begin{array}{c} (\frac{L}{PTT}{)}^{2}=\frac{hE_{0}e^{\alpha BP}}{\rho D} \end{array}$$ (5)

Rearranging Eq 5 to compute the BP, we obtain

$$\begin{array}{c} e^{\alpha BP}=\frac{L^{2}\rho D}{hE_{0}PTT^{2}} \end{array}$$ (6)

Applying the natural logarithm, we obtain

$$\begin{array}{c} \alpha BP=\text{ln}\frac{L^{2}\rho D}{hE_{0}PTT^{2}} \end{array}$$ (7)

Simplifying Eq 7, we obtain the value of BP as

$$\begin{array}{c} BP=-\frac{2}{\alpha }\text{ln}\;PTT+\frac{1}{\alpha }(\text{ln}\;\frac{\rho L^{2}D}{hE_{0}}) \end{array}$$ (8)

Eq 8 relates the blood pressure (*BP*) to the PTT and subject specific parameters (*α*, *ρ*, *L*, *D*, *h* and *E*_0_). Eq 8 can be utilized to calculate the systolic and diastolic blood pressures separately by using the systolic pulse transit time and the diastolic pulse transit time respectively [31] (Eqs 9 and 10).

$$\begin{array}{c} SBP=-\frac{2}{\alpha }\text{ln}\;PTT_{s}+\frac{1}{\alpha }(\text{ln}\;\frac{\rho L^{2}D_{s}}{hE_{0}}) \end{array}$$ (9)

$$\begin{array}{c} DBP=-\frac{2}{\alpha }\text{ln}\;PTT_{d}+\frac{1}{\alpha }(\text{ln}\;\frac{\rho L^{2}D_{d}}{hE_{0}}) \end{array}$$ (10)

where *D*_*s*_ and *D*_*d*_ are the systolic and diastolic arterial diameters respectively. The systolic and diastolic pulse transit time (*PTT*_*s*_ and *PTT*_*d*_ respectively) are computed as described in Section: Estimation of pulse transit time.

### Estimation of pulse transit time

**Fig 1:**
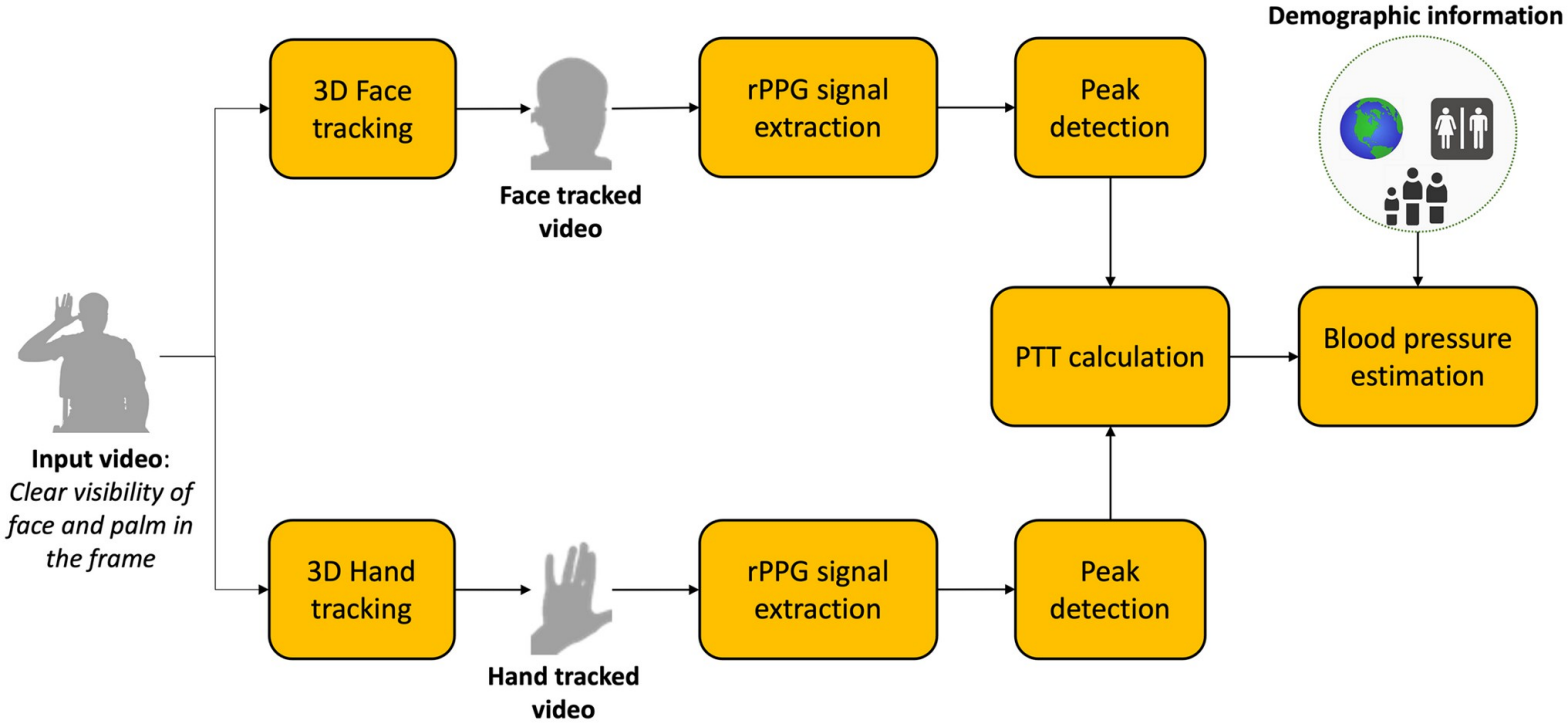
Schematic for V-BPE. Schematic diagram of V-BPE for estimating blood pressure demonstrating steps such as face/hand tracking and rPPG signal extraction.

**Fig 2:**
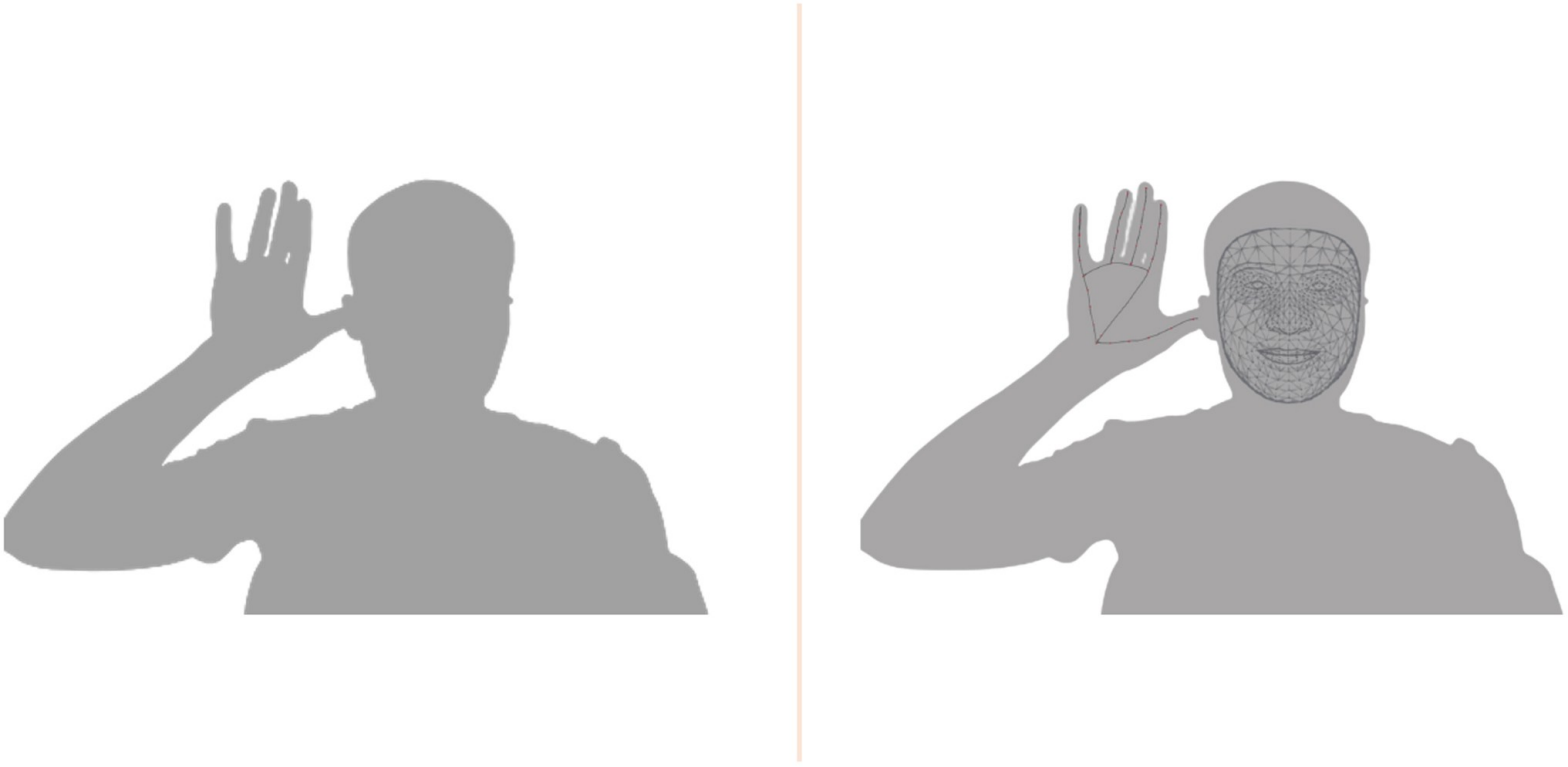
Face and Hand tracking. Subject in a seated position with right hand beside the ear with the open palm facing the camera (left). Face and hand tracking demonstrating identified landmarks on the face and the hand (right).

As depicted in Fig 1, we estimate the PTT by utilizing two rPPG signals from a single video of the face and the hand. We ensure that the subject’s face and open hand (palm facing the camera) are clearly visible in the frame of the video (Fig 2). In order to identify crucial regions of interest on the face and the hand that provide a strong PPG signal, we utilized a landmark tracking algorithm that identifies landmarks on the face and the hand throughout the duration of the video. For V-BPE, we utilize Google Mediapipe [51] as the landmark tracking approach to identify landmarks on the face and the hand. Google Mediapipe is a framework that performs real-time landmark detection and tracking on a video of a person and is robust to illumination and environmental conditions. We employed Google Mediapipe to simultaneously track landmarks on both the face and the hand given an input video (Fig 2). Google Mediapipe provides 468 landmarks on the face and 36 landmarks on the hand. We constructed regions of interest on the forehead and the palm utilizing the landmark information on the forehead and the palm, from Google Mediapipe. Moreover, the forehead and the palm are clearly visible throughout the video. The employment of a face tracking algorithm such as Google Mediapipe to construct regions of interest also reduces the influence of face and hand motion during the video. We note that V-BPE can utilize a generic face or hand tracking algorithm and is not limited to Google Mediapipe. Additionally, we note that despite differences in the arteries supplying blood to the face and hand, pulse transit time computation and the utilization of Eqs 9 and 10 is not affected [31].

**Fig 3:**
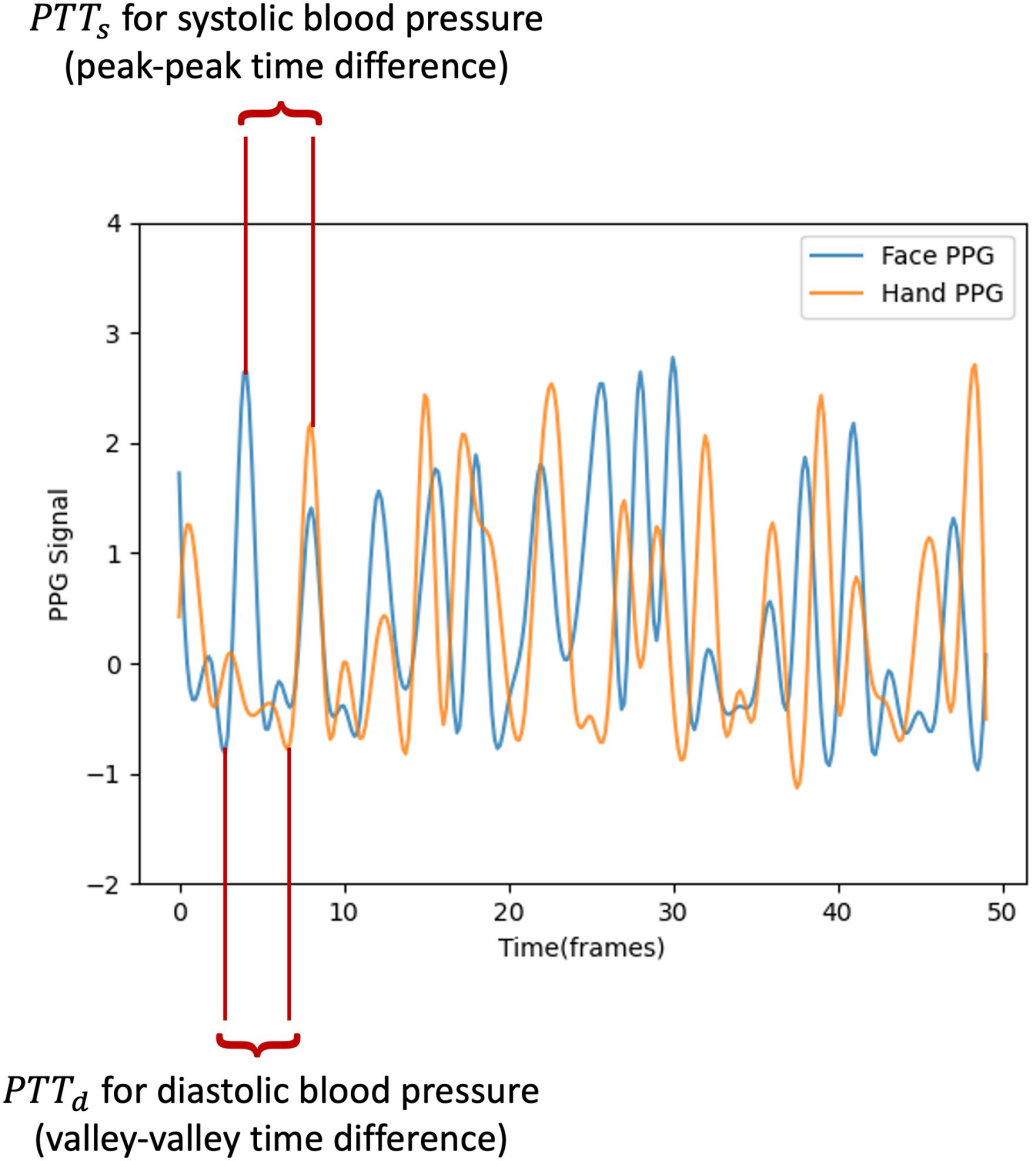
Determination of pulse transit time. Systolic pulse transit time (*PTT*_*s*_) is determined by computing the mean peak-peak distance between two PPG signals from different locations on the body. Diastolic pulse transit time (*PTT*_*d*_) is determined by computing the mean valley-valley distance between two PPG signals from different locations on the body.

A remote photoplethysmography algorithm is employed to extract the PPG signal from the constructed regions of interest. For V-BPE, we utilized the CHROM algorithm to analyze the forehead and palm regions of interest and extract the PPG signal [44]. CHROM is an open source chrominance-based method that extracts the PPG signal from a video of human skin. CHROM extracts two PPG signals (*PPG*_*Face*_ and *PPG*_*Hand*_). We filtered the obtained raw PPG signals using the butterworth bandpass filter (low cut-off frequency = 0.25 Hz, high cut-off frequency = 15 Hz) to remove low frequency noise and highlight the peaks of the signals. The peak detection algorithm [52, 53] identifies the locations of the peaks in *PPG*_*Face*_ and *PPG*_*Hand*_. We compute the time difference between each peak in *PPG*_*Face*_ and the corresponding peak in *PPG*_*Hand*_ and utilize the mean peak-peak time difference as the PTT between *PPG*_*Face*_ and *PPG*_*Hand*_ for systolic blood pressure estimation. For diastolic blood pressure estimation, we utilize the mean valley-valley time difference as the PTT between *PPG*_*Face*_ and *PPG*_*Hand*_ [31]. We compute the mean valley-valley time difference by inverting the *PPG*_*Face*_ and *PPG*_*Hand*_ signals and utilizing the peak detection algorithm (Fig 3).

### Estimation of subject specific parameters

Having obtained the PTT for systolic and diastolic blood pressure, we utilize demographic information to compute the subject specific parameters in Eqs 9 and 10 (*α*, *ρ*, *L*, *D*, *h* and *E*_0_). The utilization of demographic information is critical due to the reduced correlation between the PTT estimated from the peak/valley of the PPG signal and the SBP/DBP due to wave reflections in the late systole. Demographic features such as the height of the subject are related to the subject specific parameters such as the blood vessel length difference [54, 55].

**Fig 4:**
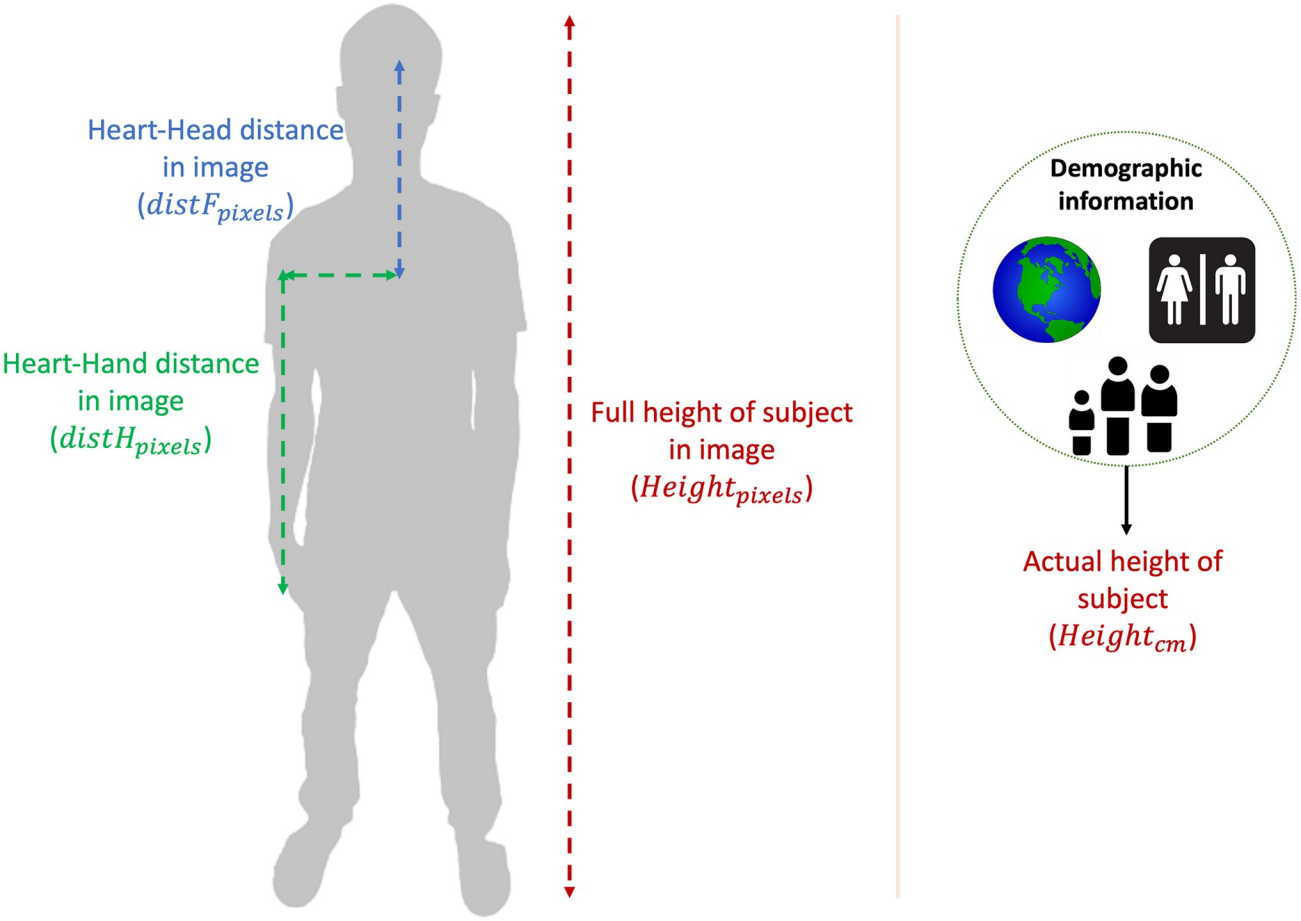
Full length image. Full length image of standing subject demonstrating the posture of the arms. The height in centimeters is extracted from the demographic data (right) and the height in pixels is identified from the full length image of the subject (left). The proportionality relation (Eq 11—with proportionality constant *p*) can be used to estimate the heart-to-hand and the heart-to-head distances.

The blood vessel length difference between the face and the hand is estimated utilizing a full length image (Fig 4) of the subject (where the subject is standing with arms straight by the side of the body as shown in Fig 4) and the height of the subject. The height of the subject is proportional to their height in the full length image [54, 56]. This can be represented as

$$\begin{array}{c} Height_{pixels}=p\times Height_{cm} \end{array}$$ (11)

where *Height*_*pixels*_ is the height of the subject in the full length image in pixels, *Height*_*cm*_ is the actual height of the subject in centimeters (measured using a medical grade height scale) and *p* is the proportionality constant. The actual height of the subject is obtained from the subject specific demographic information (Fig 4). The proportionality constant *p* incorporates the distance of the subject from the camera since the *Height*_*pixels*_ is proportional to the distance of the subject from the camera. This ensures that the mapping in Eq 11 is independent of the distance of the subject from the camera. Such independence is critical in outside-the-lab settings, where the distance of the subject from the camera is variable.

The length of the blood vessel from the heart to the face/hand is proportional to the heart to face/hand distance in pixels in the full length image [54, 56].

$$\begin{array}{c} distF_{pixels}=p\times distF_{cm} \end{array}$$ (12)

$$\begin{array}{c} distH_{pixels}=p\times distH_{cm} \end{array}$$ (13)

where *distF*_*pixels*_ and *distF*_*cm*_ are the face to heart distances in pixels and centimeters respectively and *distH*_*pixels*_ and *distH*_*cm*_ are the hand to heart distances in pixels and centimeters respectively. To estimate the proportionality constant *p*, we compute *Height*_*pixels*_ by identifying the landmarks of the person on the full length image. Utilizing the pose estimation algorithm from Google Mediapipe, we compute *Height*_*pixels*_, *distF*_*pixels*_ and *distH*_*pixels*_ (Fig 4). Having computed the *Height*_*pixels*_, we utilize the height of the person (*Height*_*cm*_) obtained from subject specific demographic information to calculate *p*. Having calculated *p*, we calculate *distF*_*cm*_ and *distH*_*cm*_ using

$$\begin{array}{c} distF_{cm}=\frac{distF_{pixels}}{p} \end{array}$$ (14)

$$\begin{array}{c} distH_{cm}=\frac{distH_{pixels}}{p} \end{array}$$ (15)

Subtracting Eq 15 from Eq 14, we estimate the blood vessel length difference (scalar difference (Fig 4) [57, 58]) between the face and the hand (*L*) as

$$\begin{array}{c} L=distF_{cm}-distH_{cm} \end{array}$$ (16)

Studies have demonstrated the relation between the parameters of the artery and the demographic parameters such as the height, age and weight of the subject [55, 59, 60] (Table 2). Kozakova et al. discovered a positive correlation between the inner radius of the artery with the weight of the subject [59]. Samijo et al. found that the age and gender of the subject are significant determinants of the artery radius [60]. Hwaung et al. established the following relation between the diameter of the artery (*D*) and demographic parameters such as age, height and weight through experimental validation (n = 231). We note that the diameter of the artery is assumed to be constant throughout the length of the artery.

$$\begin{array}{c} D=-0.258+0.029\times Height_{cm}+0.006\times Age_{years}+0.036\times \frac{Weight_{kg}}{Height_{m}^{2}} \end{array}$$ (17)

**Table 2:** Relation between the subject specific blood vessel parameters and demographic information.

Blood vessel parameter	Demographic Information	Dependence relation	Reference
Blood vessel length difference (*L*)	Height, Full length image	$\begin{array}{c} L=distF_{cm}-distH_{cm} \end{array}$	Kato and Higashiyama [54]
Diameter of artery (*D*)	Height, Age, Weight	$D=-0.258+0.029\times Height_{cm}+0.006\times Age_{years}+0.036\times \frac{Weight_{kg}}{Height_{m}^{2}}$	Hwaung et al. [55]
Artery wall thickness (*h*)	Height, Age, Weight	$h=0.25+0.005\times Age_{years}+0.005\times \frac{Weight_{kg}}{Height_{m}^{2}}$	Hwaung et al. [55]
Density of blood (*ρ*)	NA	No significant dependence on demographic parameters	Simmonds et al. [62]
Zero pressure modulus of elasticity (*E*_0_)	NA	No significant dependence on demographic parameters	Wen et al. [61]
*α*	NA	No significant dependence on demographic parameters	Ding and Zhang [29]

Hwaung et al. established the relation between the artery wall thickness with demographic parameters such as age, height and weight of the subject [55]. Through experimental validation, the following correlation was developed linking the artery wall thickness (*h*) with the age, height and weight of the subject.

$$\begin{array}{c} h=0.25+0.005\times Age_{years}+0.005\times \frac{Weight_{kg}}{Height_{m}^{2}} \end{array}$$ (18)

The density of the blood (*ρ*) and the zero pressure modulus of elasticity (*E*_0_) do not demonstrate significant variability with demographic parameters and can be regarded as constants [61, 62]. V-BPE assumes the density of blood to be 1060 *kg*/*m*^3^, the zero pressure modulus of elasticity to be 1005 and *α* to be 0.017 [29, 62].

### Experimental protocol to develop the Face-Hand dataset

The experimental setup consists of a mobile phone (Google Pixel 6) affixed to a tripod stand placed at a distance of 0.7 m from a seated subject. The phone is oriented in the landscape mode to enable the capture of a larger field of view. The mobile phone records RGB video at a frame rate of 60 fps and a resolution of 1920 × 1080 pixels. The subject is instructed to remain still and place their open right palm beside their face with the open palm facing the camera and the thumb touching the ear (Fig 2). The ground truth blood pressure information is collected using an FDA-approved cuff-based blood pressure measurement device (OMRON-BP 7000). The ground truth consists of a single value for the entire video in the SBP/DBP format. Subject height and weight are determined by medical grade height and weight scales. Informed written consent is obtained from the subject in accordance with the relevant guidelines and regulations of The Carnegie Mellon University’s *Institutional Review Board* (IRB: STUDY2019_00000362). Ethics approval for the study was also granted by the Government of Sierra Leone, Office of the Sierra Leone Ethics and Scientific Review Committee. Ethics approval for the study was also granted by Yelahanka General Hospital, Yelahanka, Bengaluru, India. Recruitment and data collection for the study was performed from May 5 2022 to October 11 2022.

The experimental protocol to record the video of the subject to create the Face-Hand dataset consists of 1) ensure the subject is seated upright facing the camera with their right hand beside their ear and facing the camera with the palm open 2) ensure the cuff-based blood pressure measurement device is affixed to the subject’s left upper arm 3) record RGB video of the subject’s face and hand in the same video frame for a duration of 120 seconds while the cuff-based device captures the ground truth blood pressure data 3) instruct the subject to stand upright with hands by their sides and facing the camera 4) capture a full length image of the standing subject 5) collect demographic information such as height, weight and age utilizing medical grade height and weight scales. The collected videos are analyzed to ensure video quality in terms of resolution, frame rate and subject posture. We collected video data from 200 subjects in outside-the-lab conditions in Sierra Leone and 200 subjects in outside-the-lab conditions in India (N = 400). The recorded videos are collected into the Face-Hand dataset and are utilized to evaluate the performance of V-BPE in outside-the-lab conditions.

### Inclusivity in global research

Additional information regarding the ethical, cultural, and scientific considerations specific to inclusivity in global research is included in the Supporting Information (S1 Questionnaire).

## Results and discussion

### Dataset

**Fig 5:**
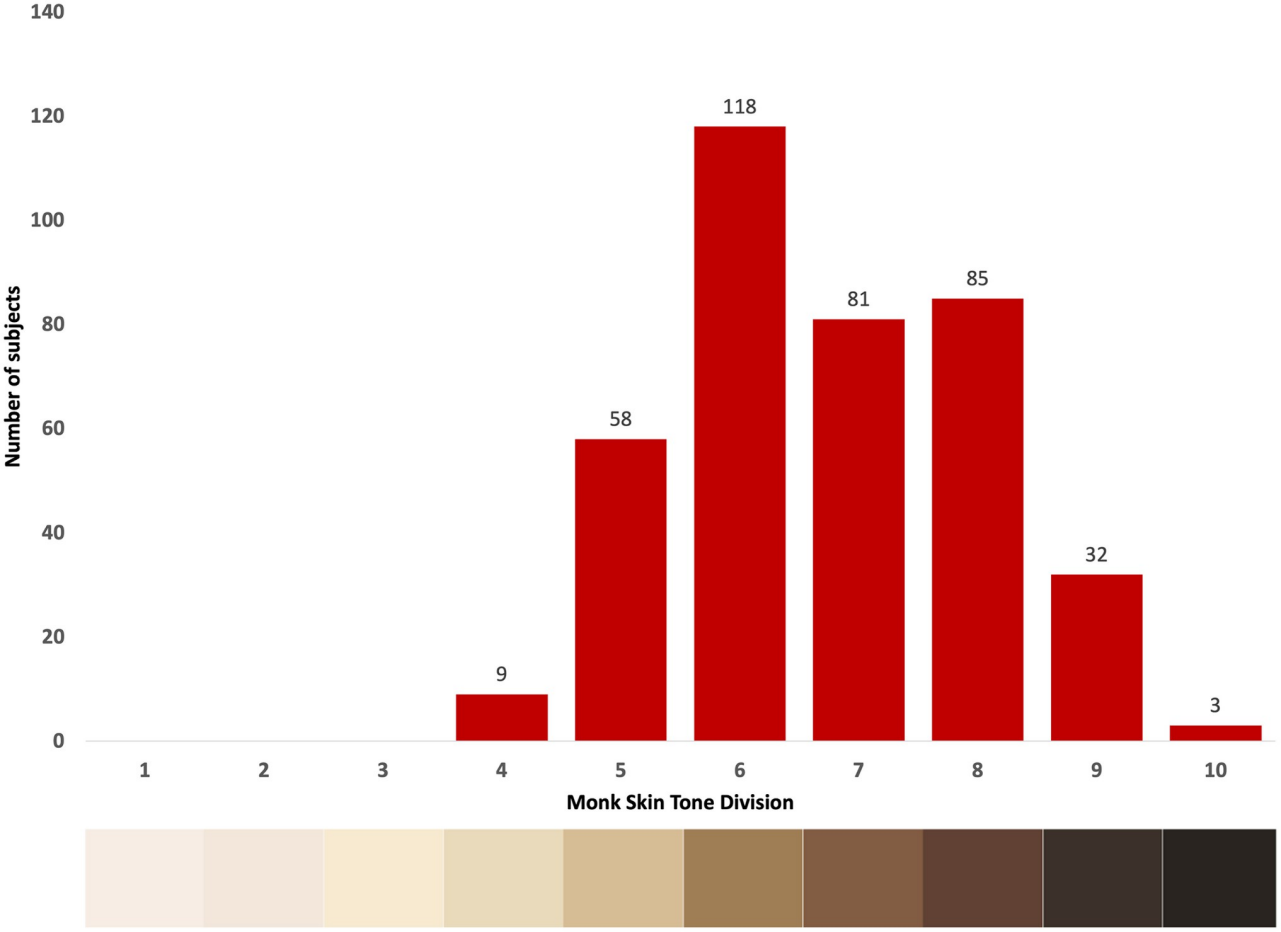
Subject skin tone distribution. Distribution of skin tone of subjects in the Face-Hand dataset according to the Monk scale [64].

The dataset used in this study (Face-Hand dataset) consists of the video and ground truth blood pressure data of 200 subjects from India and 200 subjects from Sierra Leone. The sample size of 200 subjects from each country was obtained through statistical power analysis (Statistical power = 0.9 for effect size = 0.5 (Cohen’s d [63]), no. of samples = 400, degrees of freedom = 1). The videos are recorded according to the experimental protocol described (Section: Experimental protocol to develop the Face-Hand dataset) ensuring the face and the hand are visible in the frame of the video (Fig 2). The videos in the Face-Hand dataset are recorded in an outside-the-lab environment where ambient light conditions or backgrounds are not controlled. Of the 200 subjects from India, 56 subjects are male and 144 subjects are female and of the 200 subjects from Sierra Leone, 59 subjects are male and 141 subjects are female. Additionally, the dataset contains ground truth demographic and vital sign information comprising of subject age (Mean ± Std: 40.35 ± 14.6 years; Min-Max: 17–82 years), subject height (Mean ± Std: 158.1 ± 15.1 cm; Min-Max: 68–200 cm), subject weight (Mean ± Std: 63.80 ± 14.7 kg; Min-Max: 34–160 kg), systolic blood pressure (Mean ± Std: 121.2 ± 19.7 mmHg; Min-Max: 85–202 mmHg) and diastolic blood pressure (Mean ± Std: 78.45 ± 13.3 mmHg; Min-Max: 28–126 mmHg) (Table 3). The distribution of subject skin tones is determined according to the Monk skin tone scale [64] (Fig 5). Performing the Shapiro-Wilk test, we obtain a *p*-value of 0.0475, rejecting the null hypothesis that the skin tone is normally distributed. Consequently, this result indicates that the distribution of skin tones in the Face-Hand dataset deviates from normality, underscoring the demographic diversity present within the dataset. We analyze the performance of V-BPE in outside-the-lab conditions utilizing the Face-Hand dataset, with the statistical package Scipy 1.13.0 running on Python 3.8. No data preprocessing was performed as normality of the data could not be assumed.

**Table 3:** Descriptive statistics of subjects from the Face-Hand dataset.

Group	Statistic	Age (years)	Height (cm)	Weight (kg)	SBP (mmHg)	DBP (mmHg)
**Full dataset**	Min	17	68	34	85	28
	Max	82	200	160	202	126
	Mean ± std	40.35 ± 14.6	158.1 ± 15.1	63.80 ± 14.7	121.2 ± 19.7	78.45 ± 13.3
**India**	Min	17	133	34	85	28
	Max	82	195	101	191	113
	Mean ± std	45.0 ± 14.2	157 ± 9.60	62.0 ± 11.7	118 ± 19.6	76.5 ± 13.1
**Sierra Leone**	Min	17	68	42	85	50
	Max	75	200	160	202	126
	Mean ± std	34.6 ± 12.9	159 ± 19.8	66.0 ± 17.5	125 ± 19.3	80.9 ± 13.3
**Female**	Min	17	68	34	85	47
	Max	72	191	130	202	116
	Mean ± std	39.4 ± 13.9	154 ± 15.2	62.6 ± 14.1	119 ± 20.1	78.2 ± 13.3
**Male**	Min	18	140	41	97	28
	Max	82	200	160	202	126
	Mean ± std	42.4 ± 15.9	167 ± 9.90	66.5 ± 15.7	126 ± 17.8	79.1 ± 13.5

We ensure that the videos in the Face-Hand dataset consist of a single subject, with the face and hand clearly visible in the frame of the video. The face and the hand of the subject are simultaneously tracked using a face/hand tracker that can provide dense facial and hand landmarks. We process the video to remove the frames where the hand/face is incorrectly positioned or occluded, causing a loss of hand/face tracking. Studies have demonstrated that the forehead and the cheeks contain crucial information for estimating the PPG signal from the face [65, 66] and the palm contains crucial information for PPG signal extraction from the hand [66, 67]. V-BPE extracts a region of interest using specific landmarks bounding the forehead and the palm to ensure the consistent detection of a suitably sized region of interest that scales according to the distance of the subject from the camera.

### Experimental results

V-BPE computes a single SBP/DBP value for each video in the Face-Hand dataset. We test the performance of V-BPE in challenging outside-the-lab conditions utilizing the videos from the Face-Hand dataset. We hypothesize that the estimation of the blood pressure utilizing subject specific demographic information such as age, height and weight (section: Estimation of subject specific parameters) is not significantly different from the estimation of blood pressure without using subject specific demographic information. To explore the influence of subject-specific demographic information on the performance of V-BPE, we specified fixed values for the blood vessel length difference (*L*), the radius of the artery (*r*) and the artery wall thickness (*h*) instead of utilizing the relations described in section: Estimation of subject specific parameters. According to results from existing studies [18, 68–70], *L* is assigned a value of 0.2 m, *r* is assigned a value of 0.005 m and *h* is assigned a value of 0.001 m. We estimated the blood pressure of all the subjects from the Face-Hand dataset using 1) V-BPE with *L*, *r* and *h* estimated from subject specific demographic information (denoted by Demographic-V-BPE), 2) V-BPE with fixed values for *L*, *r* and *h* (denoted by Fixed-V-BPE). We construct the null and alternate hypotheses as follows to test for the significance of including subject specific demographic information in the estimation of 1) systolic blood pressure, 2) diastolic blood pressure.

For systolic blood pressure, the null and alternate hypotheses are defined as

$$\begin{array}{c} H_{0}:SBP_{d}=SBP_{f} \end{array}$$ (19)

$$\begin{array}{c} H_{a}:SBP_{d}\neq SBP_{f} \end{array}$$ (20)

where *SBP*_*d*_ is the distribution of computed systolic blood pressure values for the videos from the Face-Hand dataset utilizing Demographic-V-BPE and *SBP*_*f*_ is the distribution of computed systolic blood pressure values for the videos from the Face-Hand dataset utilizing Fixed-V-BPE. Using power analysis, we determine a significance level of *α* = 0.05 (effect size = 0.5 (Cohen’s d [63]), no. of samples = 400, power = 0.9, degrees of freedom = 1). Performing the two sample Kolmogorov-Smirnov test, we obtain the a *p*-value of 0.00, rejecting the null hypothesis and demonstrating that there is a statistically significant difference in the SBP estimates for Demographic-V-BPE as compared to Fixed-V-BPE (Table 4).

**Table 4:** *p*-values obtained for the hypothesis tests 19–46 examining the performance of Demographic-V-BPE and Fixed-V-BPE in different demographic groups. Significant results are shaded (*α* = 0.05).

Dataset 1	Dataset 2	*p* value (*α* = 0.05)
SBP (Demographic-V-BPE)	SBP (Fixed-V-BPE)	0.00
DBP (Demographic-V-BPE)	DBP (Fixed-V-BPE)	0.00
SBP (Demographic-V-BPE)	SBP (Ground Truth)	0.20
DBP (Demographic-V-BPE)	DBP (Ground Truth)	0.30
SBP (Fixed-V-BPE)	SBP (Ground Truth)	0.00
DBP (Fixed-V-BPE)	DBP (Ground Truth)	0.00
SBP (India-Ground Truth)	SBP (Sierra Leone-Ground Truth)	0.00
DBP (India-Ground Truth)	DBP (Sierra Leone-Ground Truth)	0.03
SBP (India-Demographic-V-BPE)	SBP (Sierra Leone-Demographic-V-BPE)	0.02
DBP (India-Demographic-V-BPE)	DBP (Sierra Leone-Demographic-V-BPE)	0.01
SBP (Female-Ground Truth)	SBP (Male-Ground Truth)	0.00
DBP (Female-Ground Truth)	DBP (Male-Ground Truth)	0.64
SBP (Female-Demographic-V-BPE)	SBP (Male-Demographic-V-BPE)	0.00
DBP (Female-Demographic-V-BPE)	DBP (Male-Demographic-V-BPE)	0.16

For diastolic blood pressure, the null and alternate hypotheses are defined as

$$\begin{array}{c} H_{0}:DBP_{d}=DBP_{f} \end{array}$$ (21)

$$\begin{array}{c} H_{a}:DBP_{d}\neq DBP_{f} \end{array}$$ (22)

where *DBP*_*d*_ is the distribution of computed diastolic blood pressure values for the videos from the Face-Hand dataset utilizing Demographic-V-BPE and *DBP*_*f*_ is the distribution of computed diastolic blood pressure values for the videos from the Face-Hand dataset utilizing Fixed-V-BPE. Using power analysis, we determine a significance level of *α* = 0.05 (effect size = 0.5 (Cohen’s d [63]), no. of samples = 400, power = 0.9, degrees of freedom = 1). Performing the two sample Kolmogorov-Smirnov test, we obtain the a *p*-value of 0.00, rejecting the null hypothesis and demonstrating that there is a statistically significant difference in the DBP estimates for Demographic-V-BPE as compared to Fixed-V-BPE (Table 4).

Having observed a statistically significant difference in the SBP and DBP estimates for Demographic-V-BPE as compared to Fixed-V-BPE, we hypothesize that the estimation of SBP and DBP using Demographic-V-BPE is not significantly different from the ground truth SBP and DBP respectively.

For SBP, we construct the null and alternate hypotheses as follows for the ground truth SBP and SBP estimated by Demographic-V-BPE.

$$\begin{array}{c} H_{0}:SBP_{d}=SBP_{GT} \end{array}$$ (23)

$$\begin{array}{c} H_{a}:SBP_{d}\neq SBP_{GT} \end{array}$$ (24)

where *SBP*_*d*_ is the distribution of computed systolic blood pressure values for the videos from the Face-Hand dataset utilizing Demographic-V-BPE and *SBP*_*GT*_ is the distribution of ground truth systolic blood pressure values for the videos from the Face-Hand dataset. Using power analysis, we determine a significance level of *α* = 0.05 (effect size = 0.5 (Cohen’s d [63]), no. of samples = 400, power = 0.9, degrees of freedom = 1). Performing the two sample Kolmogorov-Smirnov test, we obtain the a *p*-value of 0.20, failing to reject the null hypothesis and demonstrating that there is no statistically significant difference in the SBP estimates for Demographic-V-BPE as compared to the ground truth SBP (Table 4).

For DBP, we construct the null and alternate hypotheses as follows for the ground truth DBP and DBP estimated by Demographic-V-BPE.

$$\begin{array}{c} H_{0}:DBP_{d}=DBP_{GT} \end{array}$$ (25)

$$\begin{array}{c} H_{a}:DBP_{d}\neq DBP_{GT} \end{array}$$ (26)

where *DBP*_*d*_ is the distribution of computed diastolic blood pressure values for the videos from the Face-Hand dataset utilizing Demographic-V-BPE and *DBP*_*GT*_ is the distribution of ground truth diastolic blood pressure values for the videos from the Face-Hand dataset. Using power analysis, we determine a significance level of *α* = 0.05 (effect size = 0.5 (Cohen’s d [63]), no. of samples = 400, power = 0.9, degrees of freedom = 1). Performing the two sample Kolmogorov-Smirnov test, we obtain the a *p*-value of 0.30, failing to reject the null hypothesis and demonstrating that there is no statistically significant difference in the DBP estimates for Demographic-V-BPE as compared to the ground truth DBP (Table 4).

Having observed no statistically significant difference for Demographic-V-BPE estimates of SBP and DBP as compared to the ground truth SBP and DBP respectively, we hypothesize that the estimation of SBP and DBP using Fixed-V-BPE is not significantly different from the ground truth SBP and DBP respectively.

For SBP, we construct the null and alternate hypotheses as follows for the ground truth SBP and SBP estimated by Fixed-V-BPE.

$$\begin{array}{c} H_{0}:SBP_{f}=SBP_{GT} \end{array}$$ (27)

$$\begin{array}{c} H_{a}:SBP_{f}\neq SBP_{GT} \end{array}$$ (28)

where *SBP*_*f*_ is the distribution of computed systolic blood pressure values for the videos from the Face-Hand dataset utilizing Fixed-V-BPE and *SBP*_*GT*_ is the distribution of ground truth systolic blood pressure values for the videos from the Face-Hand dataset. Using power analysis, we determine a significance level of *α* = 0.05 (effect size = 0.5 (Cohen’s d [63]), no. of samples = 400, power = 0.9, degrees of freedom = 1). Performing the two sample Kolmogorov-Smirnov test, we obtain the a *p*-value of 0.00, rejecting the null hypothesis and demonstrating that there is a statistically significant difference in the SBP estimates for Fixed-V-BPE as compared to the ground truth SBP (Table 4).

For DBP, we construct the null and alternate hypotheses as follows for the ground truth DBP and DBP estimated by Fixed-V-BPE.

$$\begin{array}{c} H_{0}:DBP_{f}=DBP_{GT} \end{array}$$ (29)

$$\begin{array}{c} H_{a}:DBP_{f}\neq DBP_{GT} \end{array}$$ (30)

where *DBP*_*f*_ is the distribution of computed diastolic blood pressure values for the videos from the Face-Hand dataset utilizing Fixed-V-BPE and *DBP*_*GT*_ is the distribution of ground truth diastolic blood pressure values for the videos from the Face-Hand dataset. Using power analysis, we determine a significance level of *α* = 0.05 (effect size = 0.5 (Cohen’s d [63]), no. of samples = 400, power = 0.9, degrees of freedom = 1). Performing the two sample Kolmogorov-Smirnov test, we obtain the a *p*-value of 0.00, rejecting the null hypothesis and demonstrating that there is a statistically significant difference in the DBP estimates for Fixed-V-BPE as compared to the ground truth DBP (Table 4).

**Fig 6:**
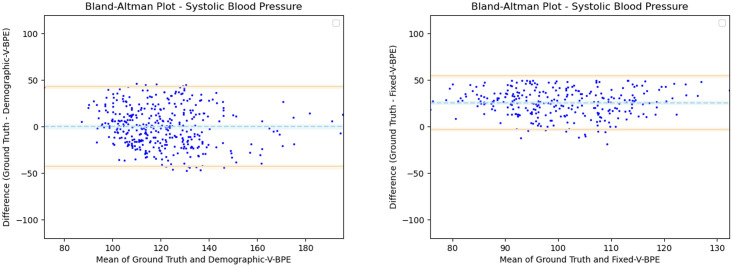
Bland Altman plots for SBP. Demographic-V-BPE estimates of SBP agree with the ground truth SBP (left), while there is significant difference in the SBP estimates of Fixed-V-BPE with the ground truth (right). Error bars and 95% confidence intervals are marked (in mmHg).

Having observed the differences in the estimation of SBP with the inclusion of subject specific demographic information, we computed the Mean Absolute Error (MAE) of the SBP estimates with respect to the ground truth SBP. V-BPE obtains an MAE of 17.1 mmHg for Demographic-V-BPE and an MAE of 36.2 mmHg for Fixed-V-BPE. The reduction of MAE on the inclusion of subject specific demographic information demonstrates the sensitivity of the systolic blood pressure to demographic differences between subjects. The Bland Altman plots (Fig 6) compare the predicted systolic blood pressure estimation with Demographic-V-BPE and Fixed-V-BPE to the ground truth. Demographic-V-BPE estimates of SBP display a mean difference of 1 mmHg, demonstrating the agreement with the ground truth SBP. In contrast, Fixed-V-BPE estimates for SBP display a mean difference of 28 mmHg with the ground truth demonstrating that Fixed-V-BPE under-estimates the systolic blood pressure. Additionally, we compute the performance (for SBP) of existing approaches for video-based blood pressure estimation utilizing the Face-Hand dataset to evaluate their robustness to various demographic groups. Table 5 demonstrates the performance of regression-based approaches (such as Secerbegovic et al. [36] and Zhou et al. [37]) and deep learning-based approaches (such as Hamoud et al. [38] and Trirongjitmoah et al. [39]) as compared to Fixed-V-BPE and Demographic-V-BPE. Demographic-V-BPE demonstrates a reduction in the MAE for SBP over different demographic groups as compared to existing regression-based and deep learning-based approaches. Regression-based and deep learning-based approaches demonstrated limited robustness to changing demographic conditions. The Bland-Altman plots for the regression-based approaches (Secerbegovic et al. [36] and Zhou et al. [37]) (Figs 8 and 9) display mean differences of -40 mmHg and -24 mmHg respectively and demonstrate that regression-based approaches over-estimate SBP. The Bland-Altman plots for the deep learning-based approaches (Hamoud et al. [38] and Trirongjitmoah et al. [39]) (Figs 10 and 11) display mean differences of 10mmHg (demonstrating under-estimation of SBP) and -45 mmHg (demonstrating over-estimation of SBP).

**Table 5:** SBP statistics (MAE and ICC) across different demographic groups for various video-based blood pressure estimation methods, compared with Demographic-V-BPE and Fixed-V-BPE.

Method	Demographic groups									
	SBP		SBP (IN)		SBP (SL)		SBP (F)		SBP (M)	
	MAE↓	ICC↑	MAE↓	ICC↑	MAE↓	ICC↑	MAE↓	ICC↑	MAE↓	ICC↑
Secerbegovic et al. [36]	38.0	0.983	35.3	0.973	41.4	0.960	35.8	0.979	43.3	0.930
Hamoud et al. [38]	23.1	0.986	23.5	0.981	22.6	0.981	23.4	0.983	22.0	0.976
Zhou et al. [37]	31.7	0.991	28.1	0.979	36.2	0.982	32.7	0.987	29.9	0.963
Trirongjitmoah et al. [39]	43.7	0.970	40.8	0.951	47.3	0.922	41.3	0.962	49.0	0.865
Fixed-V-BPE [18, 68–70]	36.2	0.914	31.9	0.902	38.2	0.891	32.3	0.909	39.5	0.860
Demographic-V-BPE	17.1	0.997	16.6	0.996	19.0	0.995	17.5	0.997	17.9	0.992

**Fig 7:**
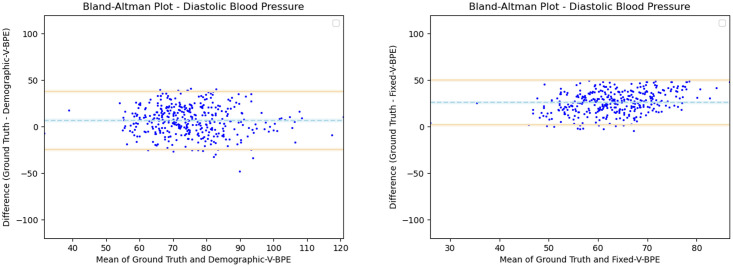
Bland Altman plots for DBP. Demographic-V-BPE estimates of DBP agree with the ground truth DBP (left), while there is significant difference in the DBP estimates of Fixed-V-BPE with the ground truth (right). Error bars and 95% confidence intervals are marked (in mmHg).

**Fig 8:**
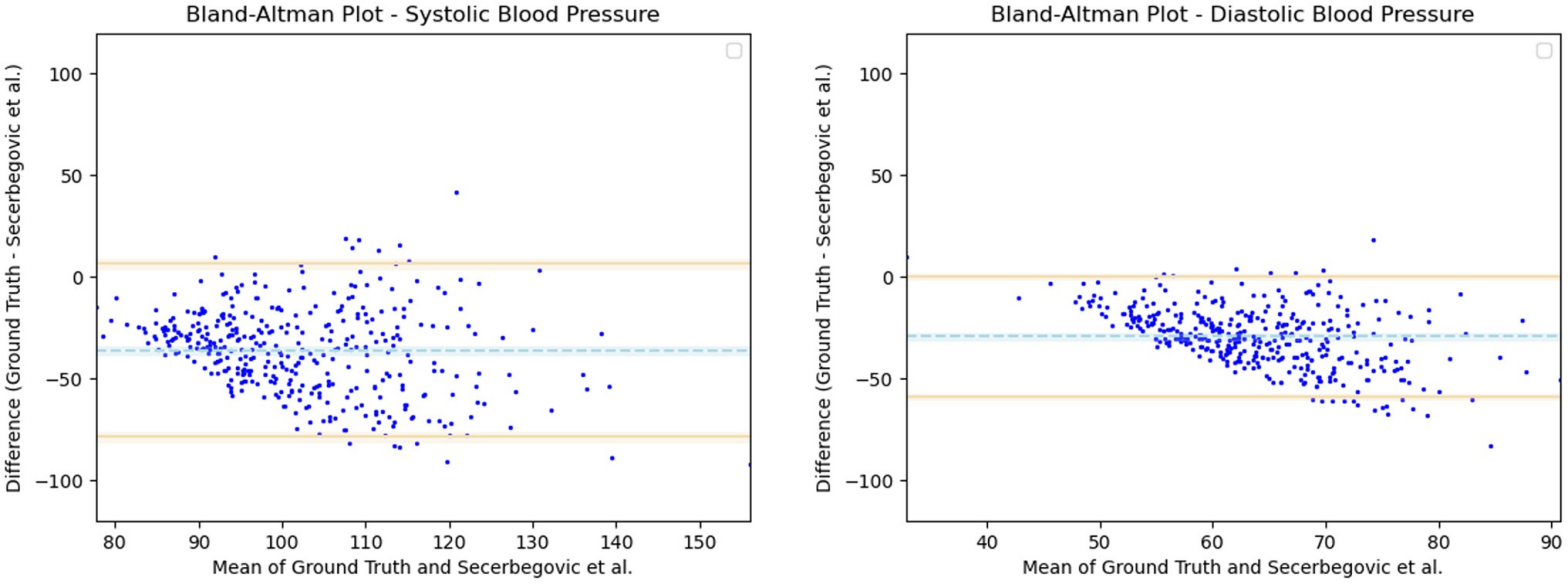
Bland Altman plots for Secerbegovic et al. [36]. SBP (left) and DBP (right) estimates utilizing Secerbegovic et al. [36] demonstrate mean differences of -40 mmHg and -30 mmHg respectively, with the ground truth. Error bars and 95% confidence intervals are marked (in mmHg).

**Fig 9:**
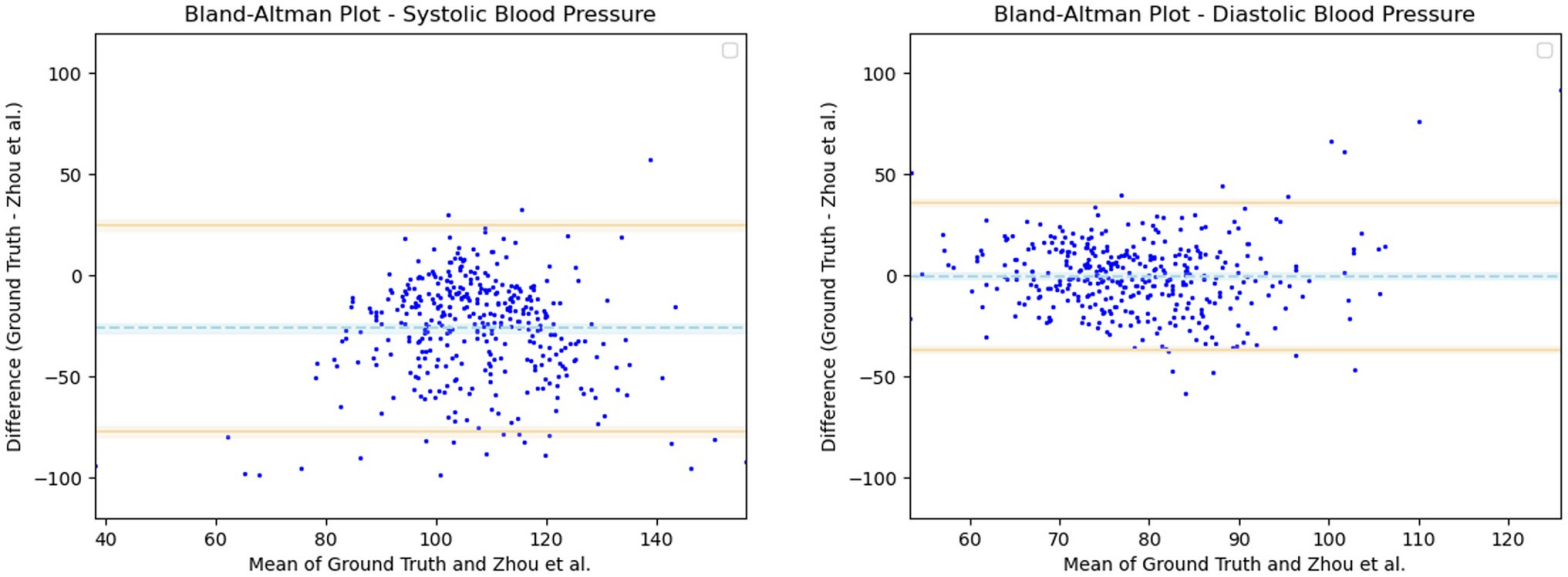
Bland Altman plots for Zhou et al. [37]. SBP (left) and DBP (right) estimates utilizing Zhou et al. [37] demonstrate mean differences of -24 mmHg and -1 mmHg respectively, with the ground truth. Error bars and 95% confidence intervals are marked (in mmHg).

**Fig 10:**
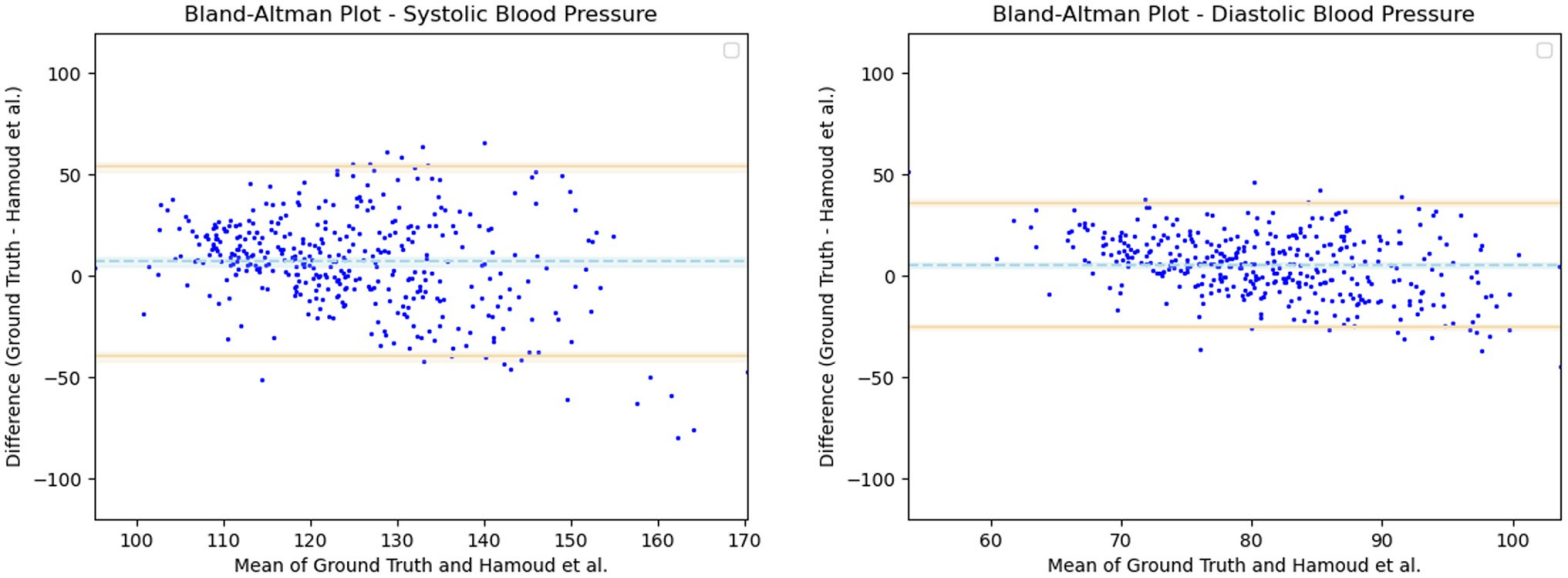
Bland Altman plots for Hamoud et al. [38]. SBP (left) and DBP (right) estimates utilizing Hamoud et al. [38] demonstrate mean differences of 10 mmHg and 10 mmHg respectively, with the ground truth. Error bars and 95% confidence intervals are marked (in mmHg).

**Fig 11:**
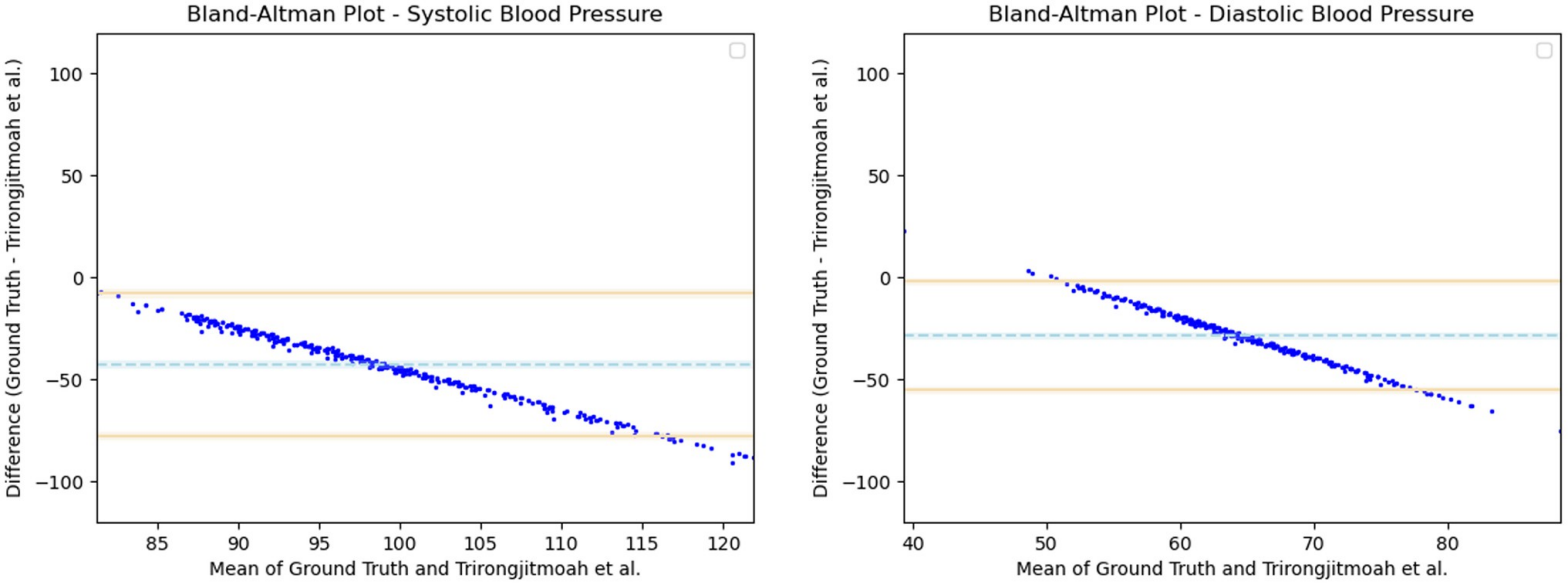
Bland Altman plots for Trirongjitmoah et al. [39]. SBP (left) and DBP (right) estimates utilizing Trirongjitmoah et al. [39] demonstrate mean differences of -45 mmHg and -28 mmHg respectively, with the ground truth. Error bars and 95% confidence intervals are marked (in mmHg).

Having observed the differences in the estimation of DBP with the inclusion of subject specific demographic information, we computed the Mean Absolute Error (MAE) of the DBP estimates with respect to the ground truth DBP. V-BPE obtains an MAE of 13.2 mmHg for Demographic-V-BPE and an MAE of 29.1 mmHg for Fixed-V-BPE. The reduction of MAE on the inclusion of subject specific demographic information demonstrates the sensitivity of the diastolic blood pressure to demographic differences between subjects. The Bland Altman plots (Fig 7) compare the predicted diastolic blood pressure estimation with Demographic-V-BPE and Fixed-V-BPE to the ground truth. Demographic-V-BPE estimates of DBP display a mean difference of 5 mmHg, demonstrating the agreement with the ground truth DBP. In contrast, Fixed-V-BPE estimates for DBP display a mean difference of 22 mmHg with the ground truth demonstrating that Fixed-V-BPE under-estimates the diastolic blood pressure. Additionally, we compute the performance (for DBP) of existing approaches for video-based blood pressure estimation utilizing the Face-Hand dataset to evaluate their robustness to various demographic groups. Table 6 demonstrates the performance of regression-based approaches (such as Secerbegovic et al. [36] and Zhou et al. [37]) and deep learning-based approaches (such as Hamoud et al. [38] and Trirongjitmoah et al. [39]) as compared to Fixed-V-BPE and Demographic-V-BPE. Demographic-V-BPE demonstrates a reduction in the MAE for DBP over different demographic groups as compared to existing regression-based and deep learning-based approaches. Regression-based and deep learning-based approaches demonstrated limited robustness to changing demographic conditions. The Bland-Altman plots for the regression-based approaches (Secerbegovic et al. [36] and Zhou et al. [37]) (Figs 8 and 9) display mean differences of -30 mmHg (demonstrating over-estimation of DBP) and -1 mmHg (demonstrating good agreement). The Bland-Altman plots for the deep learning-based approaches (Hamoud et al. [38] and Trirongjitmoah et al. [39]) (Figs 10 and 11) display mean differences of 10mmHg (demonstrating under-estimation of DBP) and -28 mmHg (demonstrating over-estimation of DBP).

**Table 6:** DBP statistics (MAE and ICC) across different demographic groups for various video-based blood pressure estimation methods, compared with Demographic-V-BPE and Fixed-V-BPE.

Method	Demographic groups									
	DBP		DBP (IN)		DBP (SL)		DBP (F)		DBP (M)	
	MAE↓	ICC↑	MAE↓	ICC↑	MAE↓	ICC↑	MAE↓	ICC↑	MAE↓	ICC↑
Secerbegovic et al. [36]	29.4	0.979	27.3	0.965	32.0	0.949	29.1	0.970	30.2	0.930
Hamoud et al. [38]	17.1	0.986	17.1	0.982	17.2	0.981	17.3	0.984	16.8	0.976
Zhou et al. [37]	16.2	0.995	11.9	0.992	21.6	0.987	17.1	0.992	14.3	0.984
Trirongjitmoah et al. [39]	28.4	0.972	26.5	0.954	30.6	0.930	28.0	0.960	29.1	0.911
Fixed-V-BPE [18, 68–70]	29.1	0.909	27.3	0.896	31.4	0.880	28.8	0.901	29.9	0.863
Demographic-V-BPE	13.2	0.997	13.4	0.995	14.2	0.993	14.0	0.995	12.8	0.992

To summarize, we note that existing video-based blood pressure estimation approaches show limited robustness in diverse demographic conditions. Moreover, we note that the estimation of SBP is more challenging that the estimation of DBP (Tables 5 and 6). Demographic-V-BPE demonstrated improved performance for both SBP and DBP estimation across different demographic groups as compared to existing approaches.

The difference in the performance of Demographic-V-BPE and Fixed-V-BPE for the estimation of systolic and diastolic blood pressure demonstrates that subject specific demographic information is critical in the estimation of blood pressure. The Face-Hand dataset contains a wide variety of subject specific demographic data such as the height, the weight, the age, the gender and the country of origin of the subject. Demographic-V-BPE utilizes the height, the weight and the age of the subject to estimate the blood pressure according to existing studies which reported a correlation between the blood pressure and the height, weight and age of the subject. We investigate whether the selection of the height, the weight and the age of the subject for blood pressure estimation using Demographic-V-BPE introduces biases (that are different from the biases existing in the ground truth) with respect to other demographic parameters such as the country of origin or the gender of the subject. To investigate whether Demographic-V-BPE introduces biases due to differences in country of origin of the subject, we explore whether the ground truth SBP and DBP distributions of subjects from India and Sierra Leone show a statistically significant difference. We construct the null and alternate hypotheses for ground truth SBP and DBP for the subjects from India and Sierra Leone as follows.

For SBP,

$$\begin{array}{c} H_{0}:SBP_{India_{GT}}=SBP_{Sierra\;Leone_{GT}} \end{array}$$ (31)

$$\begin{array}{c} H_{a}:SBP_{India_{GT}}\neq SBP_{Sierra\;Leone_{GT}} \end{array}$$ (32)

and for DBP,

$$\begin{array}{c} H_{0}:DBP_{India_{GT}}=DBP_{Sierra\;Leone_{GT}} \end{array}$$ (33)

$$\begin{array}{c} H_{a}:DBP_{India_{GT}}\neq DBP_{Sierra\;Leone_{GT}} \end{array}$$ (34)

where $SBP_{India_{GT}}$ is the distribution of ground truth SBP for the subjects from India, $SBP_{Sierra\;Leone_{GT}}$ is the distribution of ground truth SBP for the subjects from Sierra Leone, $DBP_{India_{GT}}$ is the distribution of ground truth DBP for the subjects from India and $DBP_{Sierra\;Leone_{GT}}$ is the distribution of ground truth DBP for the subjects from Sierra Leone. Using power analysis, we determine a significance level of *α* = 0.05 (effect size = 0.5 (Cohen’s d [63]), no. of samples = 200, power = 0.8, degrees of freedom = 1). Performing the two sample Kolmogorov-Smirnov test for SBP (hypothesis test 18), we obtain the a *p*-value of 0.00, rejecting the null hypothesis and demonstrating that there is significant difference in the ground truth SBP distributions for subjects from India and subjects from Sierra Leone. Performing the two sample Kolmogorov-Smirnov test for DBP (hypothesis test 20), we obtain a *p*-value of 0.03, rejecting the null hypothesis and demonstrating that there is significant difference in the ground truth DBP distributions for subjects from India and subjects from Sierra Leone (Table 4).

To investigate whether Demographic-V-BPE introduces biases due to differences in country of origin of the subject, we construct the null and alternate hypotheses for SBP and DBP as follows.

**Fig 12:**
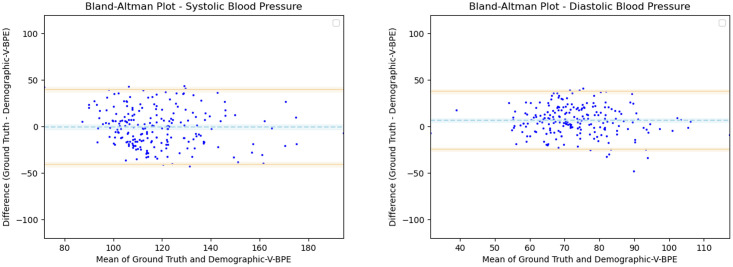
Bland Altman plots for subjects from India. Demographic-V-BPE estimates of SBP (left) as well as DBP (right) agree with the ground truth SBP and DBP respectively. Error bars and 95% confidence intervals are marked (in mmHg).

**Fig 13:**
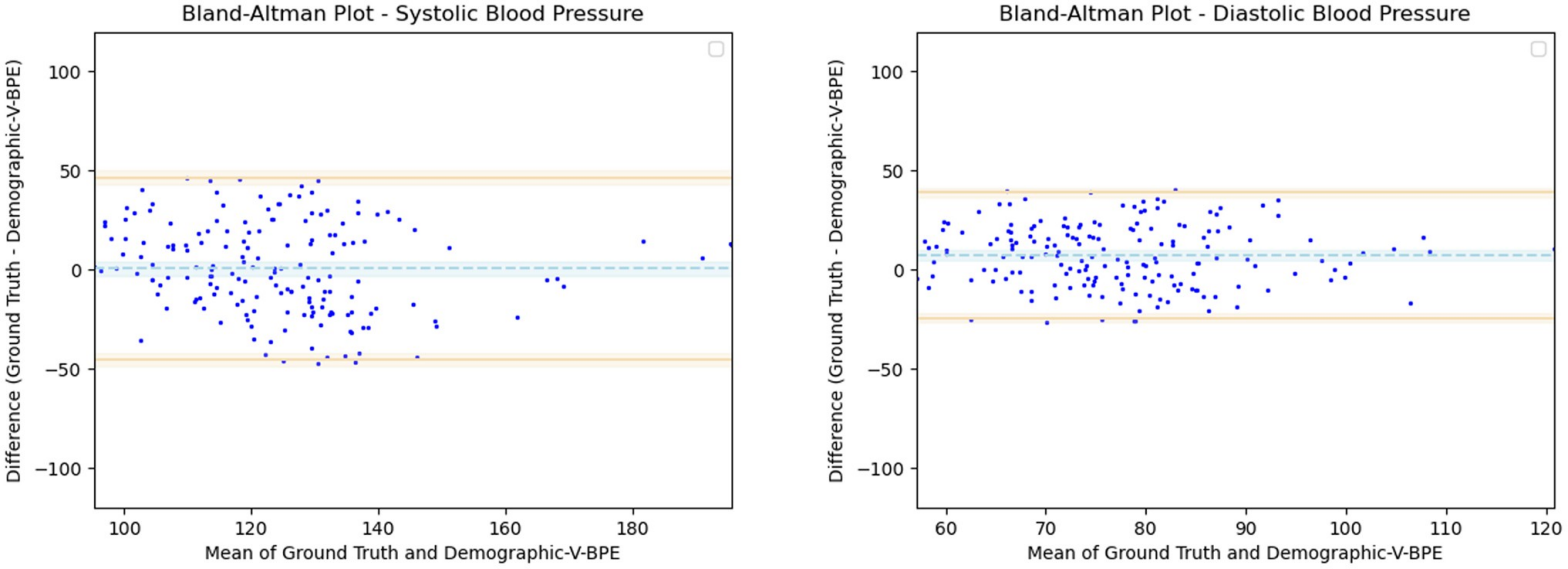
Bland Altman plots for subjects from Sierra Leone. Demographic-V-BPE estimates of SBP (left) as well as DBP (right) agree with the ground truth SBP and DBP respectively. Error bars and 95% confidence intervals are marked (in mmHg).

For SBP,

$$\begin{array}{c} H_{0}:SBP_{India_{d}}=SBP_{Sierra\;Leone_{d}} \end{array}$$ (35)

$$\begin{array}{c} H_{a}:SBP_{India_{d}}\neq SBP_{Sierra\;Leone_{d}} \end{array}$$ (36)

and for DBP,

$$\begin{array}{c} H_{0}:DBP_{India_{d}}=DBP_{Sierra\;Leone_{d}} \end{array}$$ (37)

$$\begin{array}{c} H_{a}:DBP_{India_{d}}\neq DBP_{Sierra\;Leone_{d}} \end{array}$$ (38)

where $SBP_{India_{d}}$ is the distribution of computed SBP estimates using Demographic-V-BPE for the videos of the subjects from India, $SBP_{Sierra\;Leone_{d}}$ is the distribution of computed SBP estimates using Demographic-V-BPE for the videos of the subjects from Sierra Leone, $DBP_{India_{d}}$ is the distribution of computed DBP estimates using Demographic-V-BPE for the videos of the subjects from India and $DBP_{Sierra\;Leone_{d}}$ is the distribution of computed DBP estimates using Demographic-V-BPE for the videos of the subjects from Sierra Leone. Using power analysis, we determine a significance level of *α* = 0.05. Performing the two sample Kolmogorov-Smirnov test for SBP (hypothesis test 22), we obtain the a *p*-value of 0.02 rejecting the null hypothesis and demonstrating that there is a significant difference in Demographic-V-BPE estimates of SBP for subjects from India and subjects from Sierra Leone. Performing the two sample Kolmogorov-Smirnov test for DBP (hypothesis test 24), we obtain a *p*-value of 0.01 rejecting the null hypothesis and demonstrating that there is a significant difference in Demographic-V-BPE estimates of DBP for subjects from India and subjects from Sierra Leone (Table 4). This shows that the inclusion of the height, the weight and the age in estimating blood pressure with Demographic-V-BPE does not introduce additional bias with respect to country of origin. The Bland Altman plots (Figs 12 and 13) display the agreement of the Demographic-V-BPE estimates for SBP and DBP with the ground truth SBP and DBP for India and Sierra Leone. The MAE and Inter Coder Coefficient (ICC) (Tables 5 and 6) for SBP and DBP estimates for subjects from India and Sierra Leone shows the robustness of Demographic-V-BPE in estimating blood pressure across countries.

Having explored the performance of Demographic-V-BPE across different countries, we investigate whether Demographic-V-BPE introduces biases due to differences in the gender of the subjects. We explore the distributions of the ground truth and the Demographic-V-BPE estimates for female and male subjects from the Face-Hand dataset. We construct the null and alternate hypotheses for ground truth SBP and DBP for female and male subjects as follows.

For SBP,

$$\begin{array}{c} H_{0}:SBP_{Female_{GT}}=SBP_{Male_{GT}} \end{array}$$ (39)

$$\begin{array}{c} H_{a}:SBP_{Female_{GT}}\neq SBP_{Male_{GT}} \end{array}$$ (40)

and for DBP,

$$\begin{array}{c} H_{0}:DBP_{Female_{GT}}=DBP_{Male_{GT}} \end{array}$$ (41)

$$\begin{array}{c} H_{a}:DBP_{Female_{GT}}\neq DBP_{Male_{GT}} \end{array}$$ (42)

where $SBP_{Female_{GT}}$ is the distribution of ground truth SBP for the female subjects, $SBP_{Male_{GT}}$ is the distribution of ground truth SBP for male subjects, $DBP_{Female_{GT}}$ is the distribution of ground truth DBP for the female subjects and $DBP_{Male_{GT}}$ is the distribution of ground truth DBP for male subjects. Using power analysis, we determine a significance level of *α* = 0.05 (effect size = 0.5 (Cohen’s d [63]), no. of samples = 200, power = 0.8, degrees of freedom = 1). Performing the two sample Kolmogorov-Smirnov test for SBP (hypothesis test 26), we obtain the a *p*-value of 0.00 rejecting the null hypothesis and demonstrating that there is a significant difference in the ground truth SBP distributions for female and male subjects. Performing the two sample Kolmogorov-Smirnov test for DBP (hypothesis test 28), we obtain a *p*-value of 0.64 failing to reject the null hypothesis and demonstrating that the ground truth DBP distributions for female and male subjects do not show a statistically significant difference (Table 4).

To investigate whether Demographic-V-BPE introduces biases due to differences in the gender of the subject, we construct the null and alternate hypotheses for SBP and DBP as follows.

**Fig 14:**
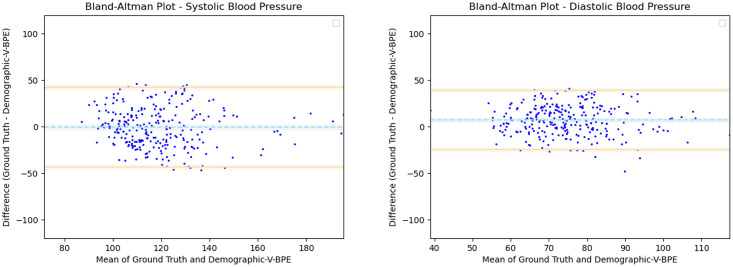
Bland Altman plots for female subjects. Demographic-V-BPE estimates of SBP (left) as well as DBP (right) agree with the ground truth SBP and DBP respectively. Error bars and 95% confidence intervals are marked (in mmHg).

**Fig 15:**
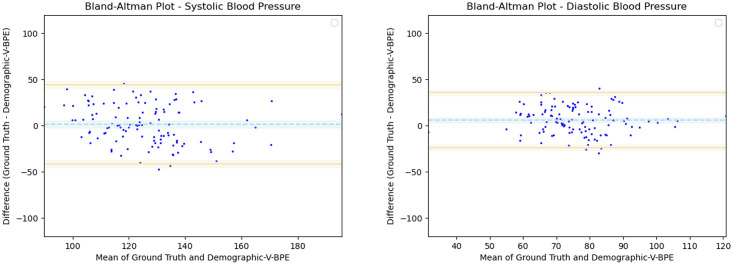
Bland Altman plots for male subjects. Bland Altman plots for female subjects. Demographic-V-BPE estimates of SBP (left) as well as DBP (right) agree with the ground truth SBP and DBP respectively. Error bars and 95% confidence intervals are marked (in mmHg).

For SBP,

$$\begin{array}{c} H_{0}:SBP_{Female_{d}}=SBP_{Male_{d}} \end{array}$$ (43)

$$\begin{array}{c} H_{a}:SBP_{Female_{d}}\neq SBP_{Male_{d}} \end{array}$$ (44)

and for DBP,

$$\begin{array}{c} H_{0}:DBP_{Female_{d}}=DBP_{Male_{d}} \end{array}$$ (45)

$$\begin{array}{c} H_{a}:DBP_{Female_{d}}\neq DBP_{Male_{d}} \end{array}$$ (46)

where $SBP_{Female_{d}}$ is the distribution of computed SBP estimates using Demographic-V-BPE for female subjects, $SBP_{Male_{d}}$ is the distribution of computed SBP estimates using Demographic-V-BPE male subjects, $DBP_{Female_{d}}$ is the distribution of computed DBP estimates using Demographic-V-BPE for female subjects and $DBP_{Male_{d}}$ is the distribution of computed DBP estimates using Demographic-V-BPE for male subjects. Using power analysis, we determine a significance level of *α* = 0.05. Performing the two sample Kolmogorov-Smirnov test for SBP (hypothesis test 30), we obtain the a *p*-value of 0.00 rejecting the null hypothesis and demonstrating that there is a significant difference in Demographic-V-BPE estimates of SBP for female and male subjects. Performing the two sample Kolmogorov-Smirnov test for DBP (hypothesis test 32), we obtain a *p*-value of 0.16 failing to reject the null hypothesis and demonstrating that Demographic-V-BPE estimates of DBP for female and male subjects do not show a statistically significant difference (Table 4). This shows that the inclusion of the height, the weight and the age in estimating blood pressure with Demographic-V-BPE does not introduce additional bias with respect to the gender of the subject. The Bland Altman plots (Figs 14 and 15) display the agreement of the Demographic-V-BPE estimates for SBP and DBP with the ground truth SBP and DBP for female and male subjects. The MAE and ICC (Tables 5 and 6) for SBP and DBP estimates for female and male subjects shows the robustness of Demographic-V-BPE in estimating blood pressure across genders.

The statistical analysis demonstrates that Demographic-V-BPE does not induce additional bias due to differences in the country of origin or the gender of subjects. In contrast, Fixed-V-BPE estimates displayed significant differences from the ground truth as well as from Demographic-V-BPE (Tables 5 and 6). The increased ICC demonstrates the agreement of SBP and DBP estimates with Demographic-V-BPE with the ground truth as compared to the SBP and DBP estimates of Fixed-V-BPE and other existing approaches with the ground truth (Tables 5 and 6). We note that the MAE for Demographic-V-BPE estimates of SBP and DBP is comparatively higher than the recommended ISO standard for BP estimation. The higher MAE is due to the demographic diversity and environmental variation in the dataset, which introduced environmental noise and demographic bias into the algorithm. However, we demonstrate that the inclusion of demographic information improves the performance of V-BPE, (Fixed-V-BPE as compared to Demographic-V-BPE) mitigating the effect of environmental noise and demographic bias. Demographic-V-BPE demonstrates improved performance as compared to other existing BP estimation approaches underscoring the importance of the inclusion of demographic information. Additionally, we note that the MAE for existing video-based BP estimation approaches is higher than the ISO standard when tested on the Face-Hand dataset highlighting the bias existing in existing approaches. We observe that the utilization of subject specific demographic information comprising of the height, the weight and the age of the subject is sufficient to inform robust blood pressure estimation. The MAE and the ICC obtained for blood pressure estimates for Demographic-V-BPE (Tables 5 and 6) demonstrates the robustness to diverse demographical distinctions.

## Conclusions

In this study, we present a an algorithm—V-BPE to estimate the systolic and diastolic blood pressure of an individual utilizing a facial and hand video of the individual, captured with a standard mobile phone camera. We utilize a pulse transit time approach to compute the blood pressure. V-BPE utilizes subject specific demographic information such as the height, weight and age to reduce the influence of demographic bias on the estimation of blood pressure. Through statistical hypothesis testing we demonstrate the significance of utilizing subject specific demographic information for blood pressure estimation. Interestingly, though we obtain an MAE outside the ISO specified range for BP estimation, we discover that the inclusion of subject specific demographic information not only improves the performance of the algorithm, but also preserves the distribution of blood pressure and does not induce additional bias. Moreover, comparative analysis with existing video-based blood pressure estimation approaches demonstrates the robustness of V-BPE. Such a result highlights the importance of subject specific demographic information in the robust estimation of blood pressure in outside-the-lab environments. Another key contribution of the study is the development of the Face-Hand dataset to test blood pressure estimation approaches. The Face-Hand dataset is curated from outside-the-lab settings and is critical in addressing common challenges associated with vital sign estimation algorithms, such as data leakage and over-constraining. Finally, the utilization of a single RGB video increases the scalability of V-BPE in resource-constrained environments due to the ubiquity of mobile phones and computers that can record RGB videos. This work provides the motivation for the development and deployment of video-based blood pressure estimation algorithms, especially in areas with limited access to resources (such as contact-based blood pressure measurement devices). Future work involves the estimation of a continuous blood pressure signal as well as a classification approach to identify subjects with hypertension and hypotension.

## Supporting information

S1 Questionnaire (DOCX)
